# BAM15 attenuates transportation-induced apoptosis in iPS-differentiated retinal tissue

**DOI:** 10.1186/s13287-019-1151-y

**Published:** 2019-02-22

**Authors:** Mingjun Tang, Ziming Luo, Yihui Wu, Jing Zhuang, Kaijing Li, Dongpeng Hu, Huifeng Rong, Bikun Xian, Jian Ge

**Affiliations:** State Key Laboratory of Ophthalmology, Guangdong Provincial Key Laboratory of Ophthalmology and Visual Science, Zhongshan Ophthalmic Center, Sun Yat-sen University, Guangzhou, Guangdong 510060 China

**Keywords:** BAM15, Apoptosis, Transportation

## Abstract

**Background:**

BAM15 is a novel mitochondrial protonophore uncoupler capable of protecting mammals from acute renal ischemic-reperfusion injury and cold-induced microtubule damage. The purpose of our study was to investigate the effect of BAM15 on apoptosis during 5-day transportation of human-induced pluripotent stem (hiPS)-differentiated retinal tissue.

**Methods:**

Retinal tissues of 30 days and 60 days were transported with or without BAM15 for 5 days in the laboratory or by real express. Immunofluorescence staining of apoptosis marker cleaved caspase3, proliferation marker Ki67, and neural axon marker NEFL was performed. And expression of apoptotic-related factors p53, NFkappaB, and TNF-a was detected by real-time PCR. Also, location of ganglion cells, photoreceptor cells, amacrine cells, and precursors of neuronal cell types in retinal tissue was stained by immunofluorescence after transportation. Furthermore, cell viability was assessed by CCK8 assay.

**Results:**

Results showed transportation remarkably intensified expression of apoptotic factor cleaved caspase3, p53, NFkappaB, and TNF-a, which could be reduced by supplement of BAM15. In addition, neurons were severely injured after transportation, with axons manifesting disrupted and tortuous by staining NEFL. And the addition of BAM15 in transportation was able to protect neuronal structure and increase cell viability without affecting subtypes cells location of retinal tissue.

**Conclusions:**

BAM15 might be used as a protective reagent on apoptosis during transporting retinal tissues, holding great potential in research and clinical applications.

**Electronic supplementary material:**

The online version of this article (10.1186/s13287-019-1151-y) contains supplementary material, which is available to authorized users.

## Background

Successful establishment of using human-induced pluripotent stem cell (hiPSC) to produce functional retinal tissues holds great potential for future therapies [1–3]. It has been a common practice to transport tissues among sites of collection, processing, and clinical areas for application [4]. However, it is reported stem cells undergo apoptosis during transportation [5]. Important considerations related to transportation include density of tissues, shipment duration, temperature management, and transportation medium [6]. Since the former three considerations have been investigated previously on other organs or stem cells [5, 7–10], we focused on seeking appropriate anti-apoptotic medium for the first time on transporting hiPSC-differentiated retinal tissues.

BAM15 is a novel mitochondrial protonophore uncoupler, which is able to transport protons into the mitochondrial matrix through a pathway without activating ATP synthase, thus uncoupling oxidative reaction from ATP production [11]. In addition, by decreasing the proton motive force across the mitochondrial inner membrane, mitochondrial uncouplers are able to accelerate the rate of mitochondrial respiration while reducing production of reactive oxygen species [12]. Therefore, the uncouplers serve as valuable and effective tools to treat myriad of diseases associated with dysregulation of oxidative phosphorylation, including obesity, aging, and neurodegeneration [13–16]. Different from the traditional uncouplers, BAM15 functions without depolarizing the plasma membrane [17, 18]. And compared to traditional uncoupler, the addition of BAM15 in cells causes less cytotoxicity and induces a comparatively higher maximum rate of mitochondrial respiration [11]. Although it has previously been reported BAM15 was capable of protecting mammals from acute renal ischemic-reperfusion injury [11] and cold-induced microtubule damage [19], the role of BAM15 in transportation remains elusive.

Precise coordination of proliferation and apoptosis is essential for retinal development and maintenance, whereas its disruption leads to abnormal structure and early onset of retinal degeneration [20]. As a cell proliferation marker, Ki-67 is a nuclear protein expressed in all cell phases except G0 [21, 22]. Apoptosis is usually initiated by the intrinsic pathway mediated by mitochondria and the extrinsic pathway mediated by cell surface death receptors [23, 24]. The two pathways converge at caspases, comprising caspase3. Thus, antibody to cleaved caspase-3 serves as a morphological marker of apoptotic cells [25]. Additionally, widely known as a tumor inhibitor, P53 is also a transcription factor that promotes apoptosis by elevating the expression of various redox-related genes [26]. And NF-κB was reported to regulate TNF-α and was associated with cells survival and apoptosis [27, 28]. TNF-α is a cytokine, which performs a pro-apoptotic role in numerous neurodegenerative disorders, and suppression of TNF-α exhibited therapeutic effects [29]. In ophthalmic disorders, TNF-α was reported to interfere with retinal detachment and apoptosis of retinal cells and leads to diabetic retinopathy [30, 31]. Thus, by detecting the above proliferation and apoptotic index, we may in this investigation confer influence of transportation on retinal apoptosis.

Also, the preservation of integral microtubule cytoskeleton is indispensible for neuronal elaborate dendritic and axonal processes as well as proper synaptic connections. Hence, failure to maintain neuronal structural integrity leads to defections in neural functions [32–34]. Neurofilament light polypeptide gene (NEFL) encodes neurofilament light polypeptide (NFL), which is one of the neuronal intermediate filaments (IFs) constructing the main cytoskeletal structure of the axon [35]. The staining of NEFL clearly manifests structure of axons [36].

Here, we report for the first time the influence of transportation on iPS-induced retinal tissue, inspiring novel pharmacological strategies to attenuate apoptosis in the process. We analyzed the appropriate conditions for transporting retinal tissue and revealed that supplement of BAM15 attenuated 5-day transportation-induced apoptosis. In addition, BAM15 was able to increase cell survival rate and protect neural structural integrity in transportation without affecting subtypes of cells’ location of retina. Prospectively, protective reagent BAM15 holds great potential for decreasing apoptosis of transportation for medical and research applications.

## Materials and methods

### Differentiation of human iPS cells into 3-D retinal cups

The hiPSC line was obtained from Cellapy® (CA4002106, Beijing, China) and maintained on Matrigel (BD Biosciences)-coated plates with StemFlex medium (ThermoFisher Scientific, MA, USA) according to WiCell protocols. The hiPS cells were passaged every 5–6 days by enzymatic digestion with 1 mM EDTA at 80% confluence. The hiPSCs were differentiated to retinal tissue based on previously established protocol with minor modifications [37, 38]. Briefly, hiPSC were dissociated into small colonies and cultured in mTeSR1 medium (STEMCELL Technologies, Vancouver, Canada) supplemented with 10 μM Blebbistatin (Sigma, USA). And this time point was defined as day 0 (D0) of differentiation. The cell clumps were cultured in suspension on D1 in the medium with mTeSR1/NIM at a 3:1 ratio and on D2 with mTeSR1/NIM at a 1:1 ratio. On D3 of differentiation, the cell aggregates were reattached on Matrigel-coated culture dishes containing NIM medium. On D16, these clusters were maintained in RDM medium and medium was changed every 2–3 days. On D28 of differentiation, horseshoe-shaped neural retina (NR) structures were identified and isolated with needle under inverted microscope. And then the NR structures were collected and transferred to RC2 medium for long-term suspension culture. The constituent of medium was reported in previous research [37]. On D30 and D60, the gradually formed three-dimensional retinal tissues were collected and subjected to experiments.

### Reagents and assay conditions

Mitochondrial uncoupler N5, N6-bis (2-Fluorophenyl)-[1,2,5] oxadiazolo [3,4-b] pyrazine-5, 6-diamine (BAM15; R&D, USA) was utilized in the assay at the concentration of 100 μM [39]. BAM15 was reconstituted in DMSO (Sigma, USA), diluted in RC2 medium, and filtrated with 0.22-μm filter before use (final DMSO concentration < 0.1%). The same amount of component solvent containing DMSO was used as a comparison in parallel in the DMSO-treated group. The retinal cups were randomly assigned to four groups in every experiment. The blank group consisted cups cultured in a humidified incubator flushed with 5% CO2 at 37 °C. Moreover, the control group contained retinal cups cultured in RC2 medium in 15-ml tube under the condition of transportation. The cups in the DMSO group and the BAM15 group were maintained in RC2 medium with DMSO and BAM15, respectively, during the process of transportation.

At the beginning of transportation, drugs were added at appropriate concentrations and transported for 5 days at room temperature on the bench without vibration, stress forces, and concessive forces to mimic transporting environment. Five days later, the cups were transferred back to culture dish in incubator at 37 °C and recovered for 5 days before subjected to experiments. To analyze actual transportation course, we used express company to deliver the retinal cups for 5 days and monitored changes of temperature by recorder (RC-4, Jingchuang Co., Ltd., China) during the process.

### Immunofluorescence staining

On day 5 after recovery, the retinal cups were fixed with 4% paraformaldehyde for 30 min, sucrose gradient dehydration and embedded in optimal cutting temperature (OCT) compound overnight at − 80 °C. The cups were sliced at 10 μm using a Leica microtome. After being permeabilized with 0.5% Triton X-100 (Sigma-Aldrich, St Louis, MO, USA) and blocked with normal goat serum (Boster, Wuhan, China), sections were stained with primary antibodies against Ki67 (Abclonal, USA), NEFL (Abclonal, USA), Laminin (Boster, Wuhan, China), and cleaved caspase-3 (CST, MA, USA) Brn3 (Santa Cruz Biotechnology, Dallas, TX) HuC/D (Santa Cruz Biotechnology, Dallas, TX) Recoverin (Abcam,Cambridge, U.K.) OTX2 (Abcam, Cambridge, U.K.) AP2α (Developmental Studies Hybridoma Bank (DSHB), Iowa, USA) PAX6 (Developmental Studies Hybridoma Bank (DSHB), Iowa, USA) at 4 °C overnight. The secondary antibodies used included the corresponding species-specific Alexa Fluor-555-, and Alexa Fluor-647-conjugated antibodies (1:500; Gibco, Carlsbad, CA) and nuclei were stained with 4′,6-diamidino-2-phenylindole (DAPI). Photos were captured with a fluorescence system (BX50; Olympus, Tokyo, Japan).

### Real-time RT-PCR

Expression of p53, NFκB, and TNF-α in retinal cups was measured after transportation by real-time PCR analyses using the SYBR Green system (TaKaRa, Tokyo, Japan). Total RNA from cups was isolated with TRIzol reagent (Sigma-Aldrich, St Louis, MO, USA). One microgram of total RNA was subjected to reverse transcription with the SYBR Prime-Script RT-PCR kit in accordance with the manufacturer’s protocol. The following primer pairs were used: for β-actin, 5′-caccacaccttctacaatgag-3′ (sense) and 5′-tagcacagcctggatagcaac-3′ (antisense); for p53, 5′-gaggttggctctgactgtacc-3′ (sense) and 5′-tccgtcccagtagattaccac-3′ (antisense); for NFκB, 5′-cgagacagtgacagtgtctgc-3′ (sense) and 5′-gctctctgagcacctttggatg-3′ (antisense); and for TNF-α, 5′-gaggccaagccctggtatg-3′ (sense) and 5′-cgggccgattgatctcagc-3′ (antisense). The experiments were repeated at least three times, and *p* value less than 0.05 was considered significant.

### Cell viability assays by CCK8

The viability of retinal cups after transportation was assessed using the Cell Counting Kit-8 (CCK8) assay (Dojindo, Kumamoto, Japan). Five days after recovery from transportation, the retinal tissues were plated into 48-well plates (3 cups/well). Each condition was performed in three replicate wells. Subsequently, the cups were incubated at 37 °C for 3 h after CCK8 reagent was added to each well. The absorbance was measured at 450 nm. The viability was calculated using the optical density ratio of a treated group relative to the control. Each set of experiments was carried out in triplicate.

### Statistical analysis

All experiments were repeated three times or more. Data are expressed as means ± SE. All calculations and statistical tests were analyzed using GraphPad Prism 6 for Mac version 4.02 (GraphPad Software, San Diego, CA) or Microsoft Excel 2003 (Microsoft, Redmond, WA). Multiple-group comparisons were carried out using one-way ANOVA. *P* value < 0.05 was considered significant.

## Results

### Influence of transportation duration and temperature on iPS-induced 3-D retinal tissues

**Fig. 1:**
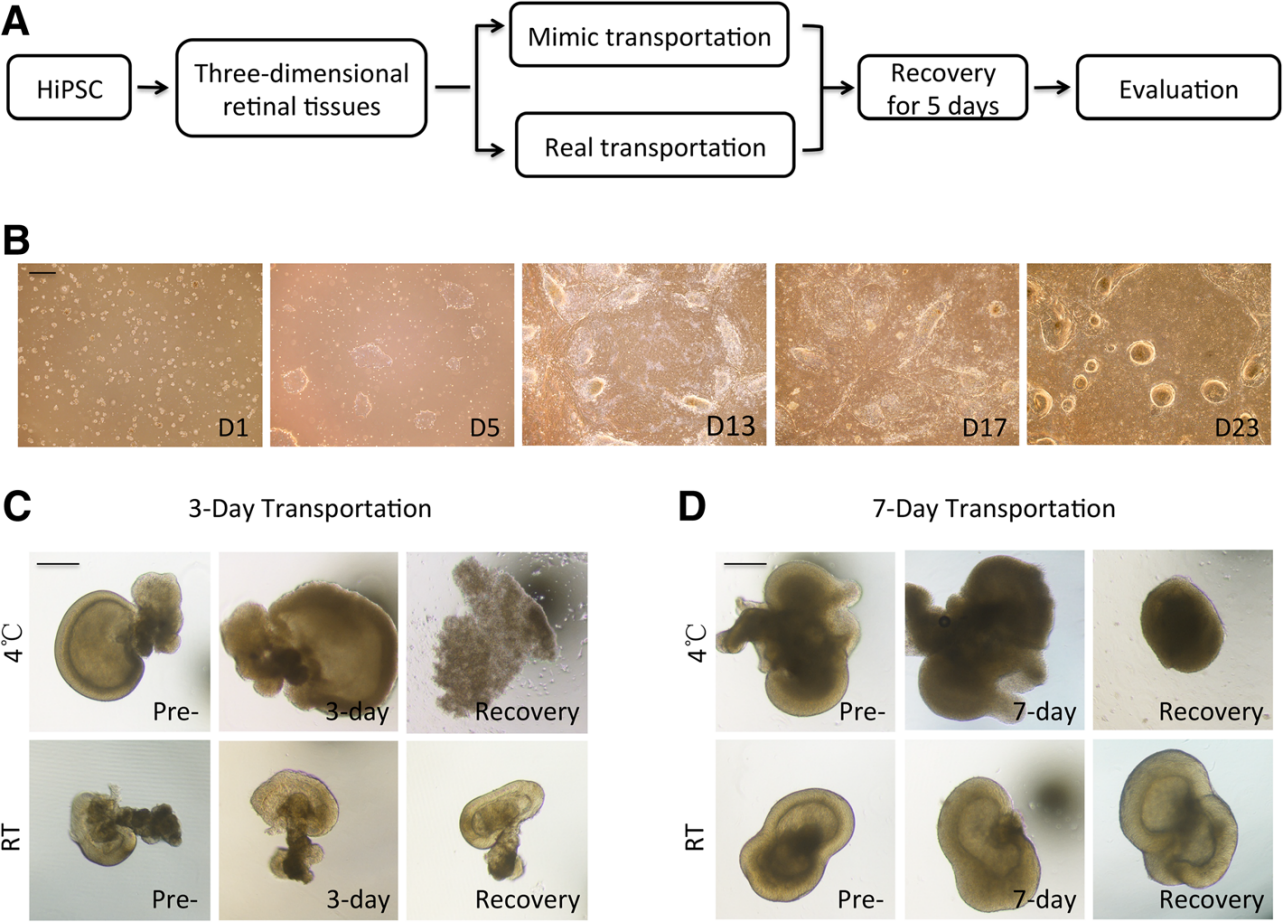
Formation of hiPSC-differentiated retinal tissue and conditions for transportation. **a** Schematic representation of experimental procedure. **b** HiPSC self-organized into eye field-like domains (EF) and subsequently differentiated into neural retina (NR), which progressively acquired an optic-cup-like shape. **c** Retinal tissue was transported for 3 days at 4 °C or room temperature. Photos were taken before transportation (Pre-), immediately after transportation (3-day), and 5 days after transportation (Recovery). **d** Photos of retinal tissue transported for 7 days were taken before transportation (Pre-), immediately after transportation (7-day), and 5 days after transportation (Recovery) at 4 °C or room temperature. Scale = 500 μm

The experimental procedures are as follows (Fig. 1a): hiPSC was differentiated into three-dimensional retinal tissues, transported by mimic transportation or by real express transportation, and evaluated after recovery for 5 days. hiPSC was differentiated into horseshoe-shaped neural retina (NR) structures (Fig. 1b), which was isolated on D28 for long-term suspension culture. Whether after 3-day transportation or 7-day transportation at 4 °C, retinal tissues manifested opaque appearance with unclear structure. In addition, tissues began to dissolve into small pieces (Fig. 1c) or shrink without original morphology (Fig. 1d) after recovery, indicating transportation at 4 °C exerts fatal influence on retinal tissues. However, no matter transported for 3 days or 7 days, retinal tissues continued to develop structure (Fig. 1c) or enlarge after recovery under room temperature transporting conditions. Since 5 days is an enough period to reach almost domestic everywhere by express, we in the subsequent experiments chose the period of 5 days for mimic transportation duration.

### Appearance of three-dimensional retinal cups before and after transportation

**Fig. 2:**
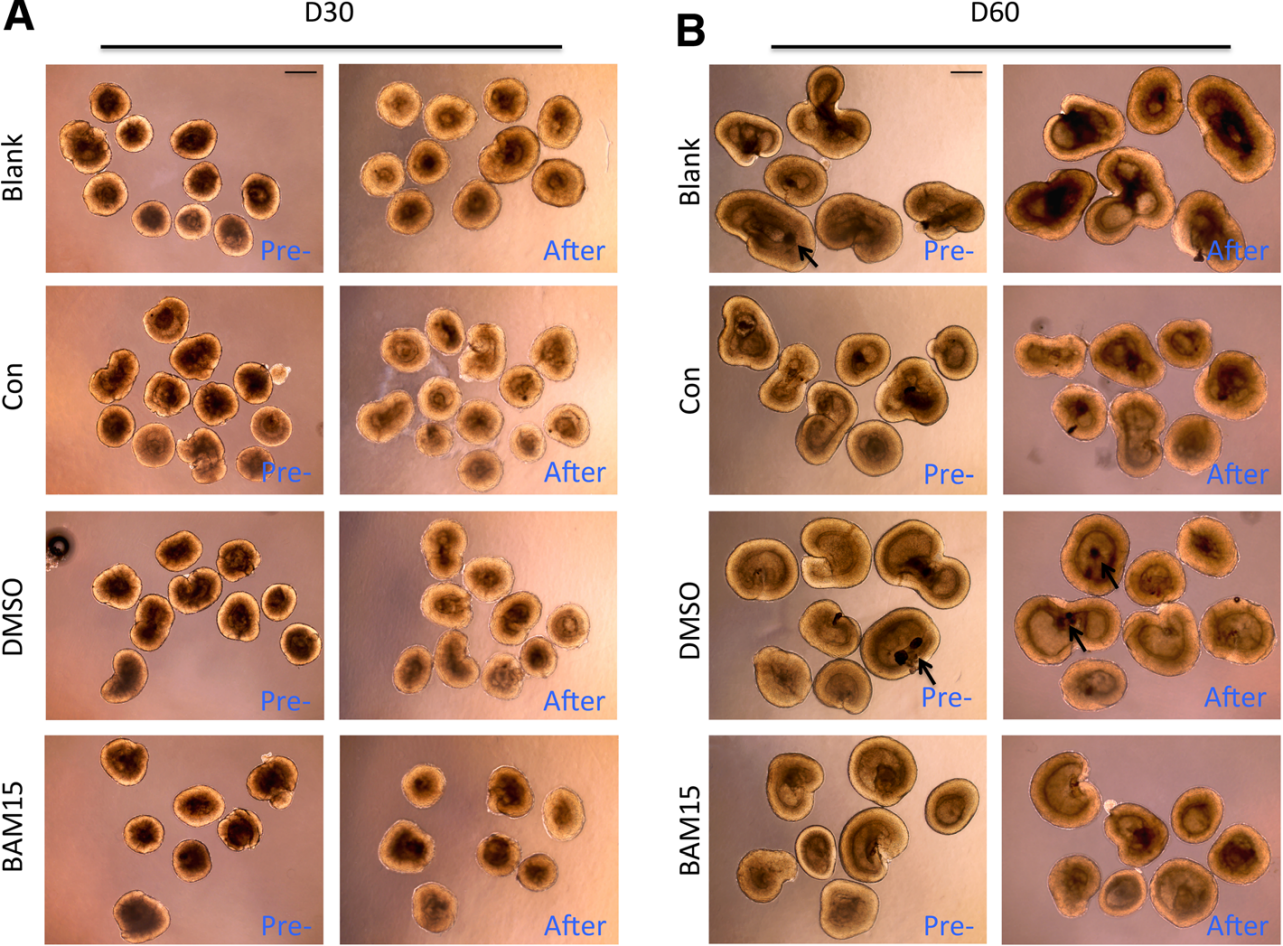
Appearance of three-dimensional retinal cups varied slightly before and after transportation. **a** Retinal cups of 30 days were transported for 5 days in different groups. Photos of cups were taken before and after transportation. **b** Photos of retinal cups on D60 were compared before and after transportation in different groups. Scale = 500 μm

As shown in Fig. 2, cups of 30 days and 60 days formed a hollow sphere, which continued to increase in size in the period of mimic transportation in four different groups. We have digested retinal cups of different size to count cell number (Additional file 1: Figure S1). According to the standard curve, average cell number of organoids at day 30 is 82,063 and at day 60 is 178,747. With or without addition of BAM15, the appearance of retinal cups changed modestly with smooth edges and clear structure before and after transportation. Similar to mimic transportation conditions, the cups in real transportation exhibited moderate changes in outward appearance. Some of the retinal cups of 60 days showed pigmented RPE (arrow) in retinal cups. And the NR resembled the historical features of actual human embryonic retina at the same age.

### Transportation increased expression of Ki67, which could be attenuated by addition of BAM15

**Fig. 3:**
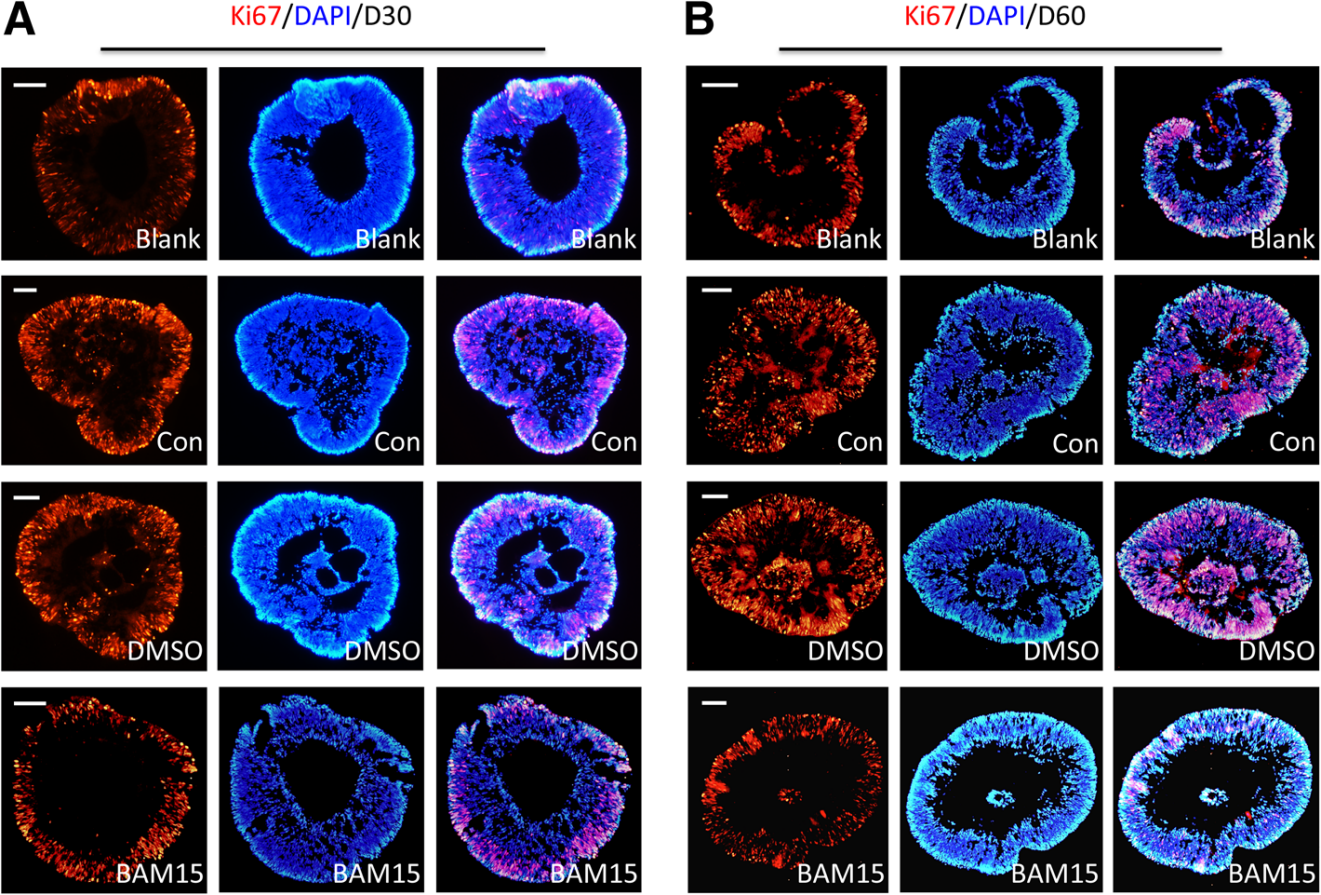
Transportation increased expression of proliferative marker Ki67, which could be attenuated by BAM15. **a** Retinal cups of 30 days manifested increase of Ki67 (Con) after 5-day transportation. And supplement of BAM15 was able to decrease expression of Ki67. **b** In both the Con and DMSO group, staining of Ki67 was intensified after transportation in retinal tissue of 60 days. The addition of BAM15 reduced staining intensity of Ki67, similar to the Blank group. Scale = 50 μm (**a**). Scale = 100 μm (**b**)

The cups presented regular and intact NR-like structures with few cavities in the blank group according to immunofluorescence staining results. While cups in the other three groups revealed loose structure and irregular cell arrangement. In the normal retinal cups without treatment, positive Ki-67 staining was observed in the outer part of the NR-like structures. Compared with the blank group, cups without BAM15 after transportation showed more nuclear staining for Ki-67, which suggested obvious proliferative stage. Moreover, numerous Ki-67 positive cells not only located in the outer part but also in the inner part of the NR-like structures after transportation without BAM15. However, with the presence of BAM15, the cups showed a significant decrease in the Ki-67^+^ cell population, which was similar to the blank group. Quantification of Ki-67^+^ was presented in Additional file 2: Figure S2A (Ki67^+^cell/1000 μm2 for D30: blank, 10.13 ± 1.19, con, 26.26 ± 2.80, DMSO, 23.17 ± 1.15, BAM15, 15.79 ± 1.58; Ki67^+^cell/1000 μm2 for D60: blank, 6.10 ± 0.53, con, 13.50 ± 0.59, DMSO, 14.16 ± 0.50, BAM15, 8.22 ± 0.25; *p* < 0.05). As a stimulating factor, transportation increased expression of Ki-67^+^ cell, which could be reduced by addition of BAM15 supplement (Fig. 3).

### BAM15 protects neuronal intactness during transportation

**Fig. 4:**
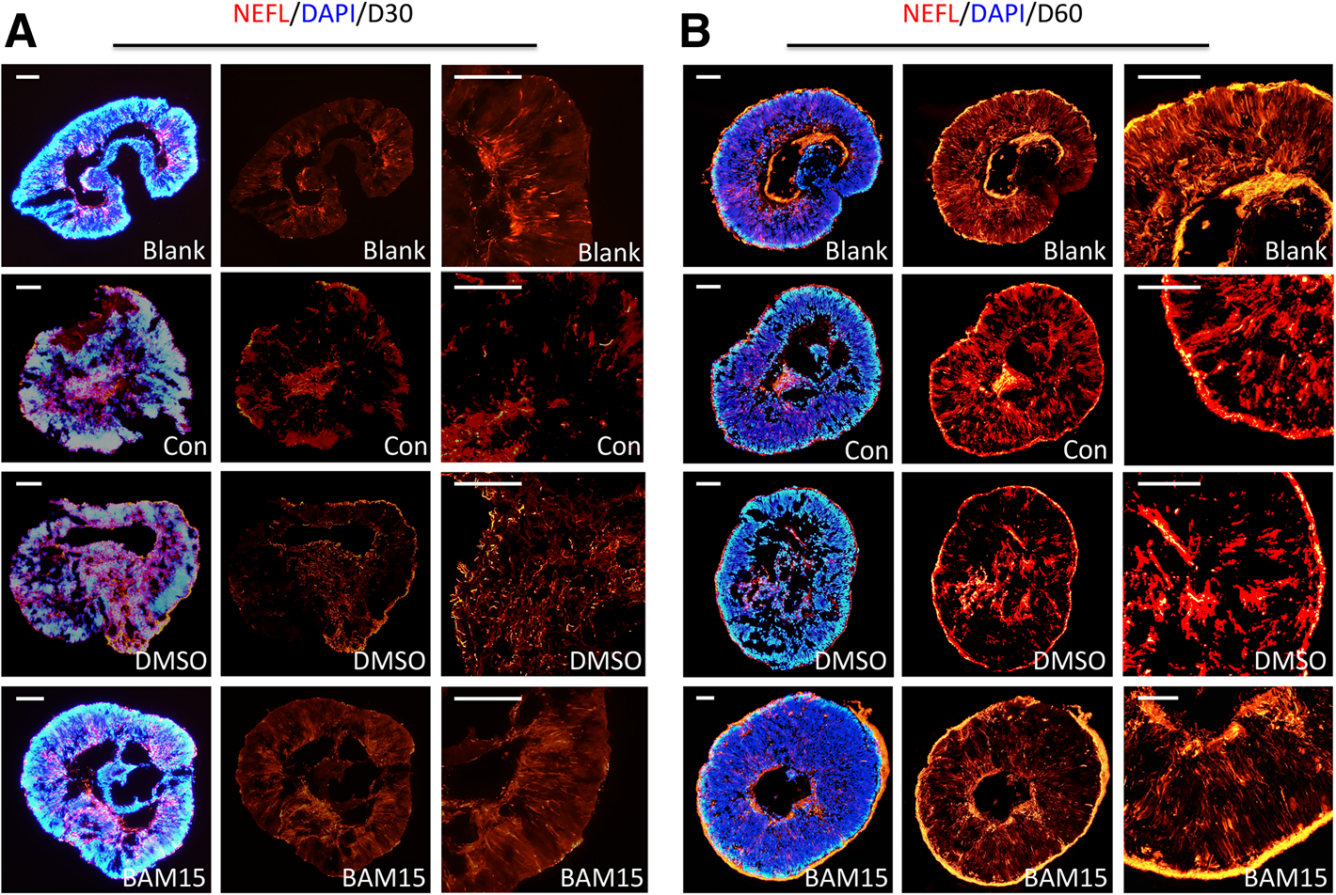
Addition of 100 μmol/L BAM15 during transportation protects neuronal intactness. **a** In cups of 30 days, immunofluorescence results revealed axons stained with NEFL appeared even and successive in the blank group. After 5 days of transportation, neurons were injured with axons manifesting disrupted in the Con group, while appeared tortuous and aggregated in the DMSO group. The use of BAM15 during transportation enables neurons to be continuous and straight. **b** In retinal tissue of 60 days, axons stained with NEFL scattered into small sections after transportation in the Con and DMSO group. And axons in the BAM15 group were longer and more continuous. Scale = 50 μm (**a**). Scale = 100 μm (**b**)

The axons stained with NEFL appeared straight and succession in cups in the blank group (Fig. 4). After transportation at room temperature without BAM15 treatment, axons became disrupted and tortuous and even congested in the center or in a certain area, especially for cups of 30 days. Compared with messed aggregation of axons in the control group, the use of BAM15 enables axons to be continuous and straight, which contributed to the intactness of neurons, thus preserving more functions of retinas. Fluorescence intensity of NEFL staining was presented in Additional file 2: Figure S2C (for D30: blank, 159.16 ± 18.33, con, 103.86 ± 5.85, DMSO, 120.53 ± 7.33, BAM15, 182.18 ± 39.94; for D60: blank, 615.43 ± 49.40, con, 389.97 ± 59.43, DMSO, 299.78 ± 35.84, BAM15, 661.58 ± 141.43).

### Transportation for 5 days increased apoptosis and deceased cell viability, and BAM15 changed this trend adversely

These immunofluorescence results indicated cleaved caspase-3 expressed occasionally in normal retinal tissues in the developing stage [37, 40–42]. In the control group after transportation, cups of 30 days and 60 days manifested a remarkably higher number of apoptotic cells. Moreover, the presence of DMSO decreased apoptosis during transportation. And the addition of BAM15 inhibited apoptosis even greater. Quantification of cleaved caspase-3 was presented in Additional file 2: Figure S2B (cleaved caspase-3^+^cell/per retinal tissue for D30: blank, 4.67 ± 0.94, con, 63.33 ± 4.78, DMSO, 48.00 ± 4.32, BAM15, 23.33 ± 3.09; cleaved caspase-3^+^cell/ per retinal tissue for D60: blank, 10.67 ± 2.05, con, 113.33 ± 10.87, DMSO, 65.33 ± 9.29, BAM15, 15.22 ± 1.63; *p* < 0.05) Thus, DMSO and BAM15 protected retinal cups from apoptosis in transportation. Laminin was mainly stained in the center of the NR-like structures and varied slightly with different treatments, suggesting BAM15 imposed little effect on expression of Laminin.

**Fig. 5:**
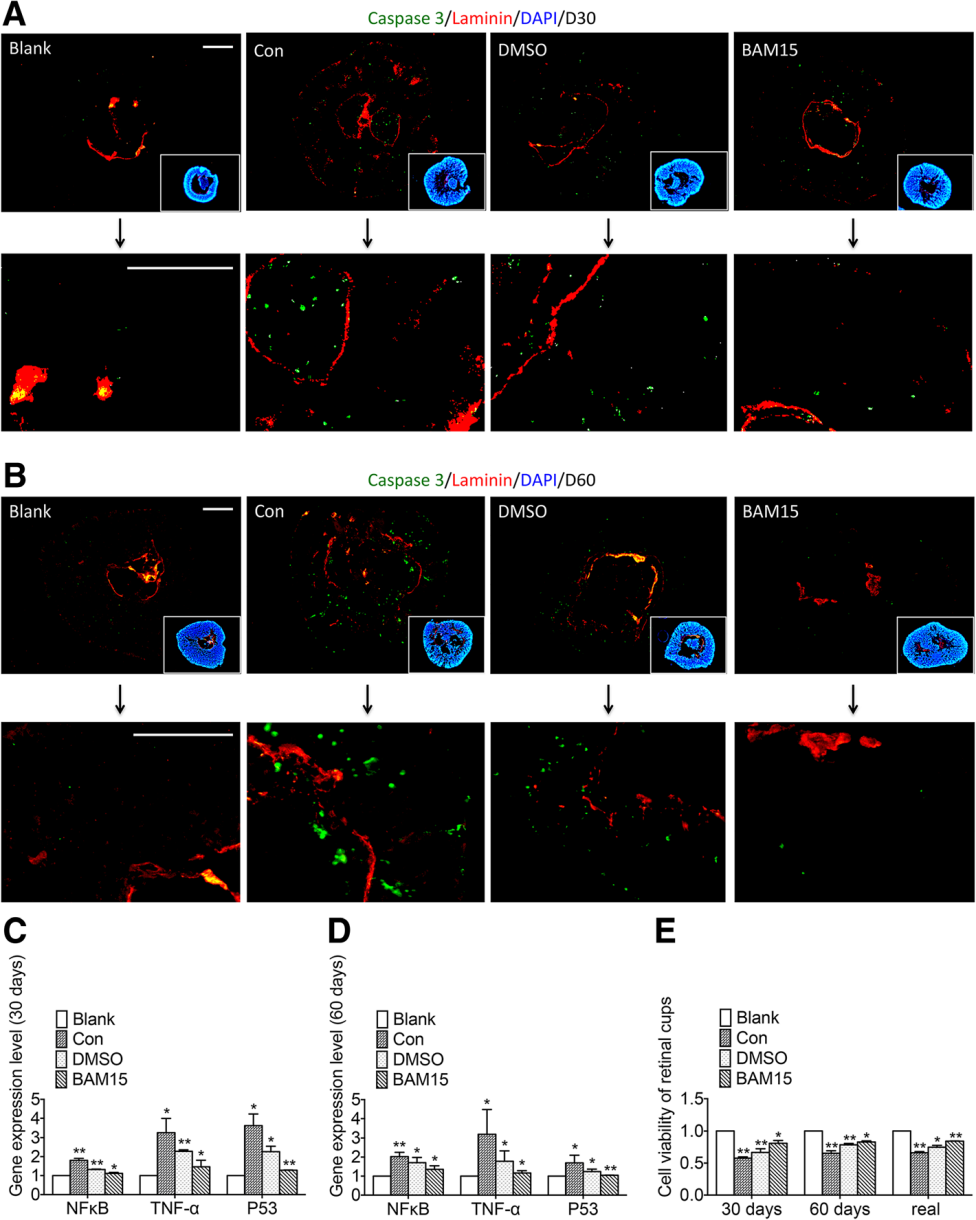
Transportation increased apoptosis and deceased cell viability, and BAM15 changed this trend adversely. **a** Immunofluorescence staining result showed transportation increased expression of Caspase-3 (green) in retinal cups of 30 days, but imposed little effects on expression of Laminin (red). The use of DMSO and BAM15 in transportation was able to alleviate the intensified expression of Caspase-3. **b** In retinal cups of 60 days, the addition of DMSO or BAM15 was able to reduce the elevated expression of Caspase-3 (green) in transportation. In addition, staining results indicated expression of Laminin (red) varied slightly in different groups. **c** Real-time PCR revealed dynamic expression changes of apoptotic-related genes NFκB, TNF-α and P53 in retinal tissue of 30 days. **d** Changes of NFκB, TNF-α, and P53 expression was assessed in retinal cups of 60 days by real-time PCR analysis. **e** Cell viability of retinal cups was assessed by CCK8 assay in transportation. Scale = 50 μm (**a**). Scale = 100 μm (**b**)

After 5 days of transportation, retinal tissue of 30 days and 60 days was extracted for real-time PCR analysis. Expression of apoptotic-related genes p53, NFkappaB, and TNF-a was elevated by transportation, and supplement of DMSO and BAM15 was able to attenuate their expression. As showed in Fig. 5c, the elevation of p53, NFkappaB, and TNF-a expression could be attenuated by supplement of DMSO, and even decreased more remarkable by the addition of BAM15 in retinal tissue of 30 days (Fig. 5c: for NFκB: blank, 1-fold, con, 1.80 ± 0.08-fold, DMSO, 1.32 ± 0.02-fold, BAM15, 1.12 ± 0.04-fold, *p* = 0.005, 0.001, 0.025 respectively; for TNF-α: blank, 1-fold, con, 3.26 ± 0.60-fold, DMSO, 2.28 ± 0.06-fold; BAM15, 1.46 ± 0.28-fold, *p* = 0.016, 0.001, 0.040 respectively; for P53: blank, 1-fold, con, 3.63 ± 0.49-fold, DMSO, 2.26 ± 0.23-fold, BAM15, 1.29 ± 0.01-fold, *p* = 0.017, 0.016, 0.001 respectively). Similarly, retinal tissue of 60 days exhibited the same trend (Fig. 5d: for NFκB: blank, 1-fold, con, 2.02 ± 0.19-fold, DMSO, 1.71 ± 0.22-fold, BAM15, 1.36 ± 0.15-fold, *p* = 0.008, 0.023, 0.042 respectively; for TNF-α: blank, 1-fold, con, 3.20 ± 1.05-fold, DMSO, 1.78 ± 0.45-fold, BAM15, 1.16 ± 0.10-fold, *p* = 0.049, 0.034, 0.040 respectively; for P53: blank, 1-fold, con, 1.69 ± 0.33-fold, DMSO, 1.24 ± 0.11-fold, BAM15, 1.04 ± 0.01-fold, p = 0.049, 0.045, 0.001, respectively). According to results of CCK8 assay (Fig. 5e), the viability of cups at 30 days and 60 days in mimic transportation situation and real transportation exhibited similar changing trends in four groups. Both addition of DMSO and BAM15 increased the survival rate of cups, and the presence of BAM15 elevated cell viability even greater (30 days: blank, 1; con, 0.577 ± 0.016; DMSO, 0.667 ± 0.047; BAM15, 0.809 ± 0.036, *p* = 0.008, 0.006, 0.037 respectively; 60 days: blank, 1; con, 0.653 ± 0.033; DMSO, 0.786 ± 0.017; BAM15, 0.829 ± 0.014, *p* = 0.001, 0.010, 0.017, respectively). Similarly, the presence of 100 μmol/L BAM15 improved cell survival in the real transportation condition (blank, 1; con, 0.660 ± 0.016; DMSO, 0.749 ± 0.023; BAM15, 0.843 ± 0.002; *p* = 0.001, 0.042, 0.016 respectively). Thus, BAM15 is clinically safe.

### Retinal cells were stained by immunofluorescence after transportation in 3-D retinal tissues

**Fig. 6:**
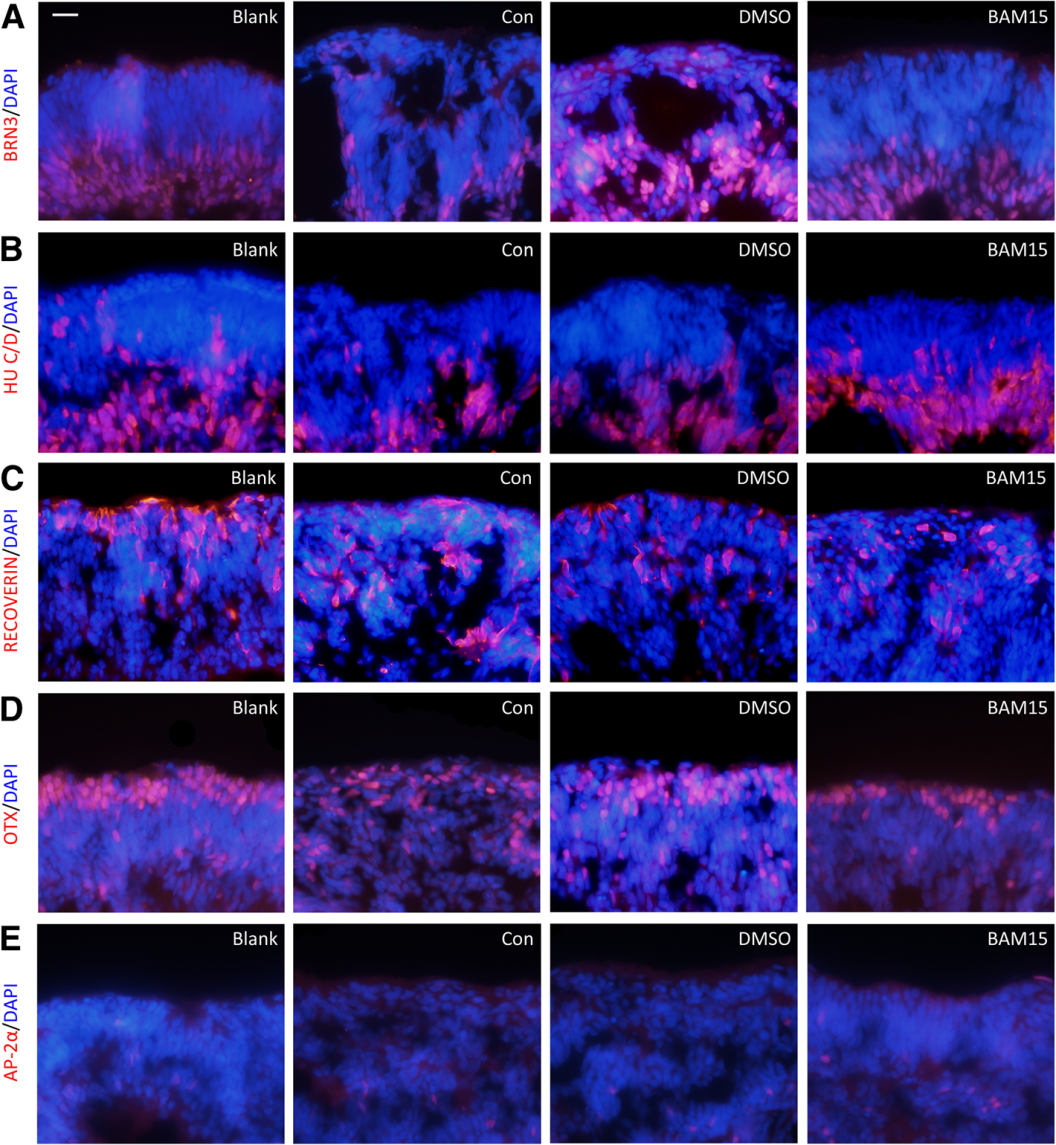
Retinal cells were stained by immunofluorescence after transportation in three-dimensional retinal tissues. Staining of ganglion cells (Brn3-/Hu C/D-positive, **a**, **b**), photoreceptor precursors (recoverin−/ OTX-positive, **c**, **d**), and amacrine cells (AP2α-positive, **e**) was presented in different groups. Scale bar = 20 μm

Similar to the results in previous investigation, hiPSC cells were able to replicate fetal retinal development and generate tissues with retinal cells in specific layers [43]. After recovery from transportation, retinal tissues Additional file 8 were around weeks 9–10; gangling cells stained with Brn3 (Fig. 6a) and HuC/D (Fig. 6b) appeared in the inner layer. And photoreceptor precursors stained with recoverin (Fig. 6c) and OTX (Fig. 6d) lined up in the outermost layer at this stage. Additionally, amacrine cells were detected by staining AP2α in Fig. 6e, and expression of PAX6 was also detected (Additional file 2: Figure S2D). Although in the control and DMSO group, the discontinuous structure of retinal tissues was apparent, subtypes of retinal cells located in the same layer as in the blank group. Statistical testing was performed, and differences between groups were not significant by quantifying these retinal cell markers (Fig. 3c: Brn3^+^cell/1000 μm^2^: blank, 7.70 ± 0.23, con, 7.32 ± 0.38, DMSO, 8.50 ± 0.42, BAM15, 7.06 ± 0.50; Hu C/D^+^ cell/1000 μm^2^: blank, 6.21 ± 0.78, con, 6.04 ± 0.17, DMSO, 5.58 ± 0.26, BAM15, 6.87 ± 0.19; recoverin^+^ cell/1000 μm^2^: blank, 3.26 ± 0.29, con, 3.35 ± 0.29, DMSO, 2.49 ± 0.25, BAM15, 3.25 ± 0.37; OTX^+^ cell/1000 μm^2^: blank, 5.24 ± 0.18, con, 5.50 ± 0.25, DMSO, 5.28 ± 0.10, BAM15, 5.32 ± 0.26; AP2α^+^cell/1000 μm^2^: blank, 0.63 ± 0.06, con, 0.49 ± 0.04, DMSO, 0.52 ± 0.01, BAM15, 0.59 ± 0.02; PAX^+^ cell/1000 μm^2^: blank, 8.03 ± 0.15, con, 8.59 ± 0.46, DMSO, 7.79 ± 0.31, BAM15, 8.46 ± 0.13). Thus, 5 days of transportation imposed little influence on location of subtypes of retinal cells. To find out whether transportation has delayed effects on differentiation, retinal tissues after 15 days of recovery from transportation were detected and similar results were observed (Additional file 3: Figure S4&S5). Statistical testing was performed, and differences between groups were not significant. Furthermore, it could be inferred from immunofluorescence results the addition of BAM15 decreased the area of disconnection in retinal tissues, contributing to structural integrity.

### In real transportation, retinal cups of 60 days showed the same trends of changes similar to mimic transportation

To investigate whether mimic transportation could really represent real transportation by express, we delivered 3-D retinal tissues by express and compared relevant index. Not surprisingly, relevant index detected in real transportation was consistent with mimic conditions. As shown in Additional file 4: Figure S6A, there were hardly any changes in an outward appearance before and after transportation in two separate groups. Besides, just as what we found in mimic transportation, the use of BAM15 decreased expression of proliferative marker Ki67 (Additional file 4: Figure S6B), protected neural integrity (Additional file 4: Figure S6C), and decreased expression of apoptotic marker cleaved-caspase 3 (Additional file 4: Figure S6D). Also, no matter in the control or BAM15-treated group, location of gangling cells (Additional file 4: Figure S6E&F), photoreceptor precursors (Additional file 4: Figure S6G&H), and amacrine cells (Additional file 4: Figure S6I) was in accordance with previous staining results without transportation. The box used in real express transportation and temperature figure was showed in Additional file 5: Figure S7. And temperature during real transportation fluctuated, but roughly around 25 °C. Therefore, the results of mimic transportation are able to represent real transportation by express.

## Discussion

In the current study, we investigated for the first time effects of 5 days transportation on hiPSC-differentiated retinal tissues. We provided novel findings on several points: (1) transportation increased apoptosis in retinal tissues; (2) transportation damaged neural structural integrity; and (3) mitochondrial uncoupler BAM15 was able to attenuate apoptosis, increase cell viability and protect neural structural integrity during transportation.

Although protocols adopted for shipment of viable cells varied among laboratories, the main consideration is to attenuate apoptosis, protect morphological intactness, and preserve cell viability [6, 10]. Filling the transporting container with ample medium and leaving as little vacant space as possible to reduce possible vibration during shipment is an important consideration [44]. In this investigation, we set 5 days as time of shipment. As to the temperature regulation, low temperature weakened temperature-dependent chemical bonds in cytoskeletal support, thus leading the cell more vulnerable to mechanical damage [6]. It also indicated that the majority of fresh blood products and sterile BM samples benefitted from transportation at room temperature [7, 8]. Similarly, low temperature is not favorable for shipment of retinal tissues in our study.

BAM15 has a broad range of efficacy without toxicity [11, 45, 46]. Similarly, our investigation proved in standard culture conditions, different concentration of BAM15 imposed little effect on cell viability slightly (Additional file 6: Figure S3A and Figure S3B), expression of NEFL (Additional file 7: Figure S8A) and expression of cleaved caspase-3 (Additional file 7: Figure S8B). Thus, BAM15 is a safe supplementary for transportation.

BAM15 might attenuate transportation-induced apoptosis by targeting relevant signaling pathways. The mitochondrial membrane regulates expression of caspase pathway, and previous evidence indicated caspase-3 and caspase-9 participated in iPSC apoptosis [47]. Also, our results showed transportation increased expression of cleaved caspase-3, which could be attenuated by adding mitochondrial uncoupler BAM15. Moreover, the investigation revealed that activation of p53 in certain cell types induced apoptosis or the cessation of cell growth [48]. And our results also showed the elevated expression of p53 in transportation could be inhibited by the treatment of BAM15. In addition, NF-κB in inflammatory cells regulates the production of chemokines and pro-inflammatory cytokines including TNF-α, and it also controls cell survival and proliferation [49, 50]. In our study, transportation increased both expressions of NF-κB and TNF-α, which could be decreased by BAM15. Therefore, we hypothesize transportation initiated several apoptotic reactions and BAM15 may function by targeting the above signaling pathways.

Five days of transportation imposed little effect on expression of laminin. Laminin presents in the developing human eyes and plays an important role in ocular differentiation [51]. In addition, laminin regulates cell death and survival and is of critical importance in neural retina and retinal pigmented epithelium [52]. What is more, previous investigation manifested laminin is associated closely with retinal ganglion cells, neuronal maturation and maintenance, photoreceptor production, differentiation, development and synaptic organization. However, our results demonstrated 5 days of transportation affects expression of laminin slightly, indicating laminin may not be involved in apoptosis during transportation.

Surprisingly, in our investigation, elevation expression of proliferative marker Ki67 was found to co-occur with increased apoptotic-related markers. The increased expression of proliferation marker was in response to stimulation of transportation, manifesting proliferation potential of stem cells was maintained during transportation. In BAM15-treated group, we surmised supplement of BAM15 reduced the influence of transportation, and thus staining of Ki67+ cells was less than the control group.

Previous investigation revealed that addition of BAM15 greatly enhanced tubulin preservation in the cold possibly by reducing mitochondrial stress [19]. Similarly, even during transportation at room temperature, our results demonstrated mitochondrial uncoupler BAM15 was able to protect neural structural integrity in retinal tissue. Thus, we hypothesized that transportation might induce neuron structural damage via mitochondrial pathway, which needed to be further explored.

In the subsequent work, the full mechanism of how BAM15 functions remains to be further explored. Moreover, we dedicated to investigate influence of BAM15 on retinal development and differentiation in the future.

## Conclusions

Addition of mitochondrial uncoupler BAM15 is capable of attenuating apoptosis, protecting neural structural integrity and increasing cell viability in 5-day transportation of three-dimensional retinal tissue.

## Additional files

Additional file 1: **Figure S1.** Relationship between area and cell number in three-dimensional retinal tissue. *X*-axis stands for area of organoid (μm2) and *y*-axis stands for cell number. (TIF 11557 kb)

Additional file 2: **Figure S2.** (A) Quantification of Ki67 in Fig. 3 (Ki67+ cell/1000 μm2 for D30: blank, 10.13 ± 1.19, con, 26.26 ± 2.80, DMSO, 23.17 ± 1.15, BAM15, 15.79 ± 1.58; Ki67+ cell/1000 μm2 for D60: blank, 6.10 ± 0.53, con, 13.50 ± 0.59, DMSO, 14.16 ± 0.50, BAM15, 8.22 ± 0.25). (B) Quantification of cleaved caspase-3 in Fig. 5 (cleaved caspase-3+ cell/per retinal tissue for D30: blank, 4.67 ± 0.94, con, 63.33 ± 4.78, DMSO, 48.00 ± 4.32, BAM15, 23.33 ± 3.09; cleaved caspase-3+ cell/ per retinal tissue for D60: blank, 10.67 ± 2.05, con, 113.33 ± 10.87, DMSO, 65.33 ± 9.29, BAM15, 15.22 ± 1.63). (C) Fluorescence intensity of NEFL staining in Fig. 4 (for D30: blank, 159.16 ± 18.33, con, 103.86 ± 5.85, DMSO, 120.53 ± 7.33, BAM15, 182.18 ± 39.94; for D60: blank, 615.43 ± 49.40, con, 389.97 ± 59.43, DMSO, 299.78 ± 35.84, BAM15, 661.58 ± 141.43). (D) Five days of transportation imposes little effect on expression of PAX6 in retinal tissue at D30 and D60. (TIF 7125 kb)

Additional file 3: **Figure S4.** Retinal cells were stained by immunofluorescence after 15 days of recovery in three-dimensional retinal tissues. Staining of ganglion cells (Brn3-/Hu C/D-positive, A-B), photoreceptor precursors (recoverin−/ OTX-positive, C-D), amacrine cells (AP2α-positive, E) was presented in different groups. (TIF 11747 kb)

Additional file 4: **Figure S6.** In real transportation, trend of changes was similar to mimic transportation. (A) In the Con group and BAM15 group, morphology of retinal tissue varied slightly before and after transportation. (B-D) Immunofluorescence results presented the staining of Caspase-3 (B), NEFL(C) and Ki67(D) after real transportation. (E-I) Staining of ganglion cells (Brn3-/Hu C/D-positive, E-F), photoreceptor precursors (recoverin−/ OTX-positive, G-H), amacrine cells (AP2α-positive, I) was presented in the Con and BAM15 group. Scale bar = 50 μm (TIF 54539 kb)

Additional file 5: **Figure S7.** The box used in real express transportation (A) and temperature at different time point during real transportation (B). (TIF 2677 kb)

Additional file 6: **Figure S3.** Effects of BAM15 on cell proliferation and differentiation. (A) Outward appearance of retinal tissue with concentration at 20 μmol/L, 50 μmol/L and 100 μmol/L BAM15 in incubator at different time point. (B) BAM15 imposes little effects on cell proliferation in retinal organoid (blank, 1; con, 0.973 ± 0.139; DMSO, 1.122 ± 0.245; BAM15, 1.032 ± 0.180; *p* value > 0.5). (C) Quantification of retinal cell marker after transportation in Fig. 6 and Additional file 2: Figure S2D (Brn3+ cell/1000 μm^2^: blank, 7.70 ± 0.23, con, 7.32 ± 0.38, DMSO, 8.50 ± 0.42, BAM15, 7.06 ± 0.50; Hu C/D+ cell/1000 μm^2^: blank, 6.21 ± 0.78, con, 6.04 ± 0.17, DMSO, 5.58 ± 0.26, BAM15, 6.87 ± 0.19; recoverin+ cell/1000 μm^2^: blank, 3.26 ± 0.29, con, 3.35 ± 0.29, DMSO, 2.49 ± 0.25, BAM15, 3.25 ± 0.37; OTX+ cell/1000 μm^2^: blank, 5.24 ± 0.18, con, 5.50 ± 0.25, DMSO, 5.28 ± 0.10, BAM15, 5.32 ± 0.26; AP2α + cell/1000 μm^2^: blank, 0.63 ± 0.06, con, 0.49 ± 0.04, DMSO, 0.52 ± 0.01, BAM15, 0.59 ± 0.02; PAX+ cell/1000 μm^2^: blank, 8.03 ± 0.15, con, 8.59 ± 0.46, DMSO, 7.79 ± 0.31, BAM15, 8.46 ± 0.13). (TIF 7512 kb)

Additional file 7: **Figure S8.** Compared with the blank group, BAM15 at 20 μmol/L, 50 μmol/L and 100 μmol/L concentration imposes little effect on NEFL (A) and cleaved caspase-3 (B) expression in the incubator as negative control. (TIF 6721 kb)

Additional file 8: **Figure S5.** (A) Outward appearance of retinal tissue after 5 days of transportation and 15 days of recovery. (B) Cell viability of retinal tissues after 5 days of transportation and 15 days of recovery (blank, 1; con, 1.02 ± 0.09; DMSO, 1.04 ± 0.05; BAM15, 1.05 ± 0.06; *p* = 0.40, 0.40, 0.37 respectively). (TIF 7675 kb)
