# Supplementary figures and images for: BAM15 attenuates transportation-induced apoptosis in iPS-differentiated retinal tissue
Source: Stem Cell Res Ther. 2019 Feb 22;10:64. doi: 10.1186/s13287-019-1151-y (PMC6387563; doi:10.1186/s13287-019-1151-y)

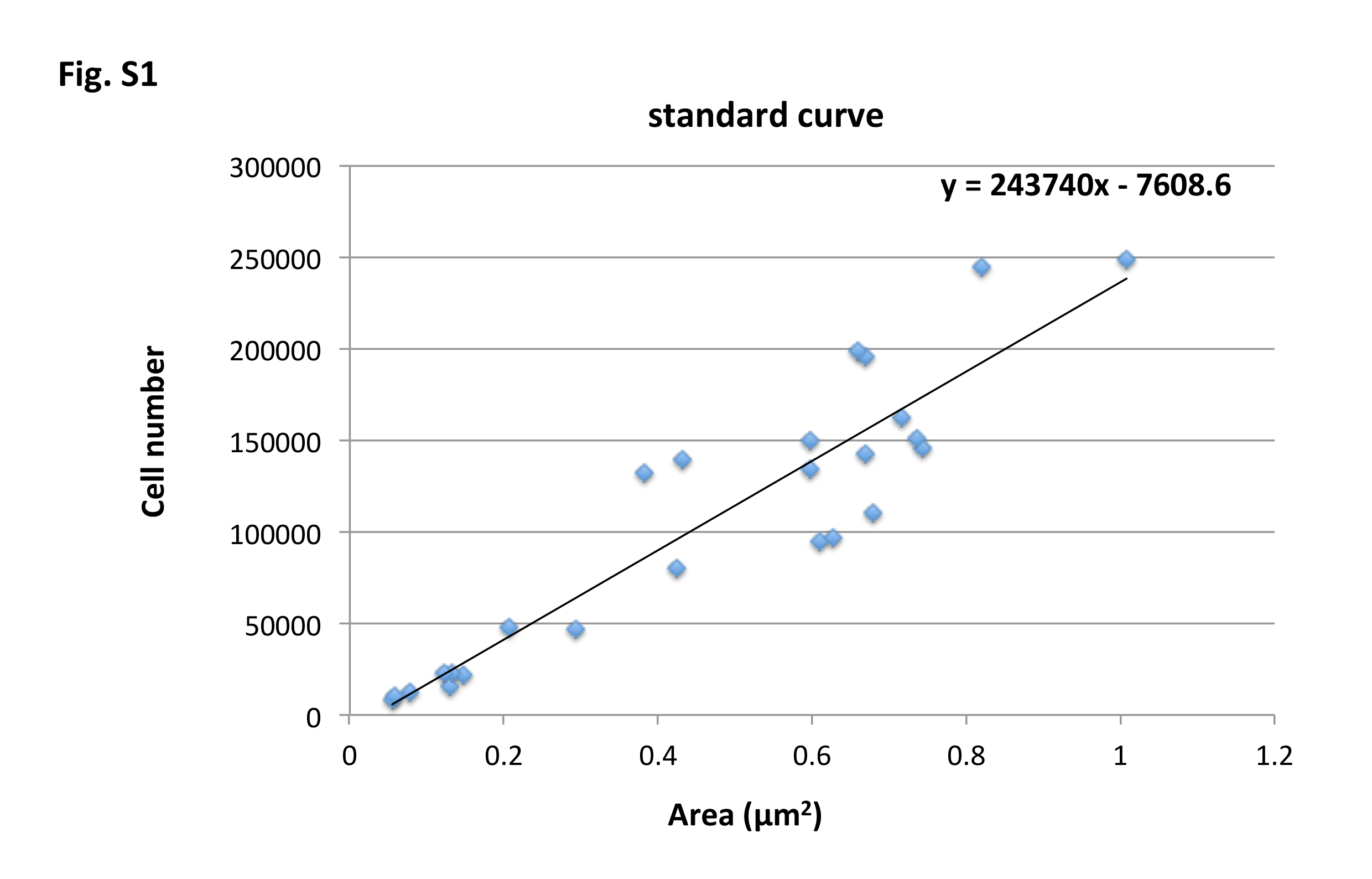

Supplement: Supplementary file 1 — Figure S1. Relationship between area and cell number in three-dimensional retinal tissue. X-axis stands for area of organoid (μm2) and y-axis stands for cell number. (TIF 11557 kb) [file 13287_2019_1151_MOESM1_ESM.tif]

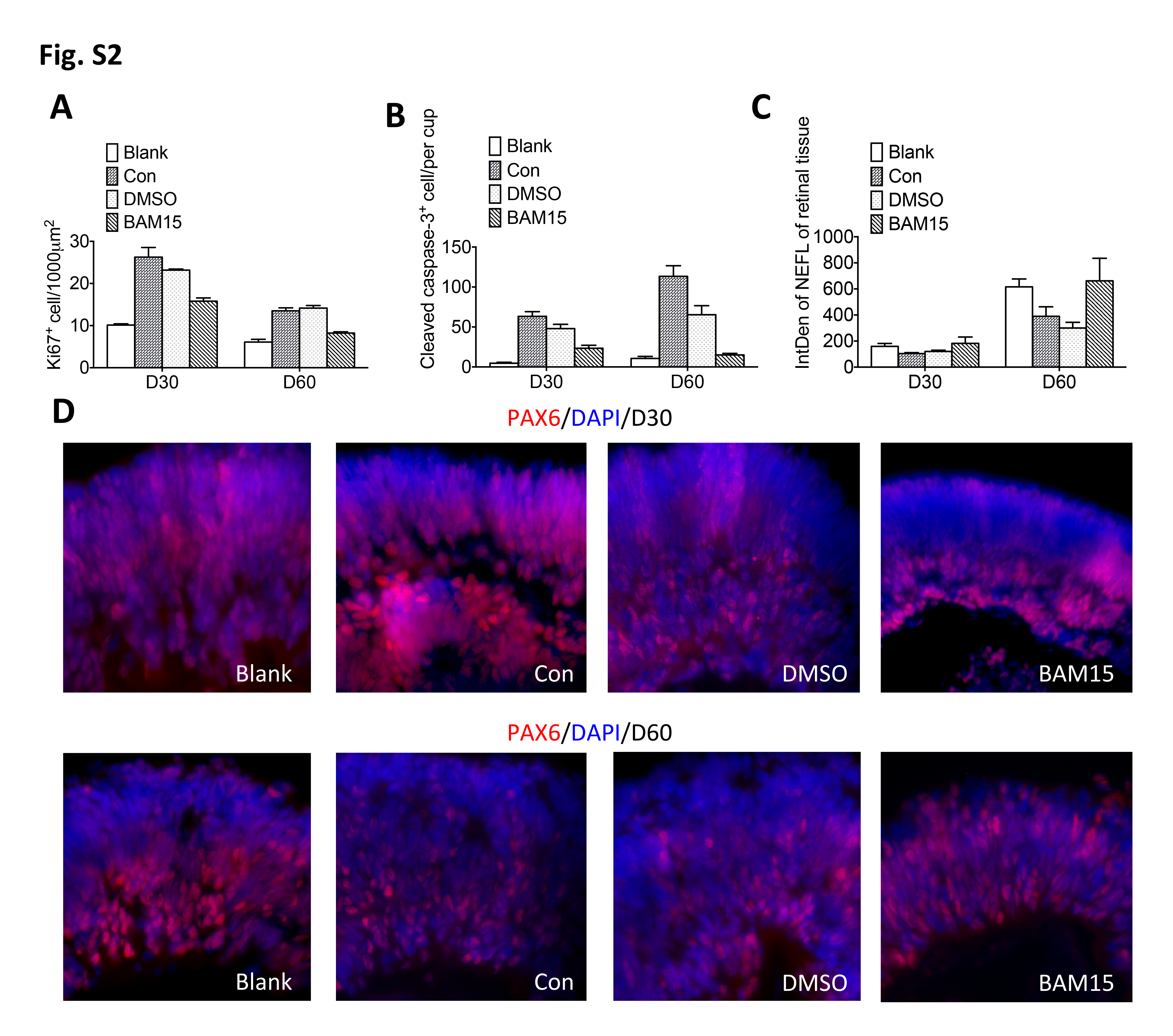

Supplement: Supplementary file 2 — Figure S2. (A) Quantification of Ki67 in Fig. 3 (Ki67+ cell/1000 μm2 for D30: blank, 10.13 ± 1.19, con, 26.26 ± 2.80, DMSO, 23.17 ± 1.15, BAM15, 15.79 ± 1.58; Ki67+ cell/1000 μm2 for D60: blank, 6.10 ± 0.53, con, 13.50 ± 0.59, DMSO, 14.16 ± 0.50, BAM15, 8.22 ± 0.25). (B) Quantification of cleaved caspase-3 in Fig. 5 (cleaved caspase-3+ cell/per retinal tissue for D30: blank, 4.67 ± 0.94, con, 63.33 ± 4.78, DMSO, 48.00 ± 4.32, BAM15, 23.33 ± 3.09; cleaved caspase-3+ cell/ per retinal tissue for D60: blank, 10.67 ± 2.05, con, 113.33 ± 10.87, DMSO, 65.33 ± 9.29, BAM15, 15.22 ± 1.63). (C) Fluorescence intensity of NEFL staining in Fig. 4 (for D30: blank, 159.16 ± 18.33, con, 103.86 ± 5.85, DMSO, 120.53 ± 7.33, BAM15, 182.18 ± 39.94; for D60: blank, 615.43 ± 49.40, con, 389.97 ± 59.43, DMSO, 299.78 ± 35.84, BAM15, 661.58 ± 141.43). (D) Five days of transportation imposes little effect on expression of PAX6 in retinal tissue at D30 and D60. (TIF 7125 kb) [file 13287_2019_1151_MOESM2_ESM.tif]

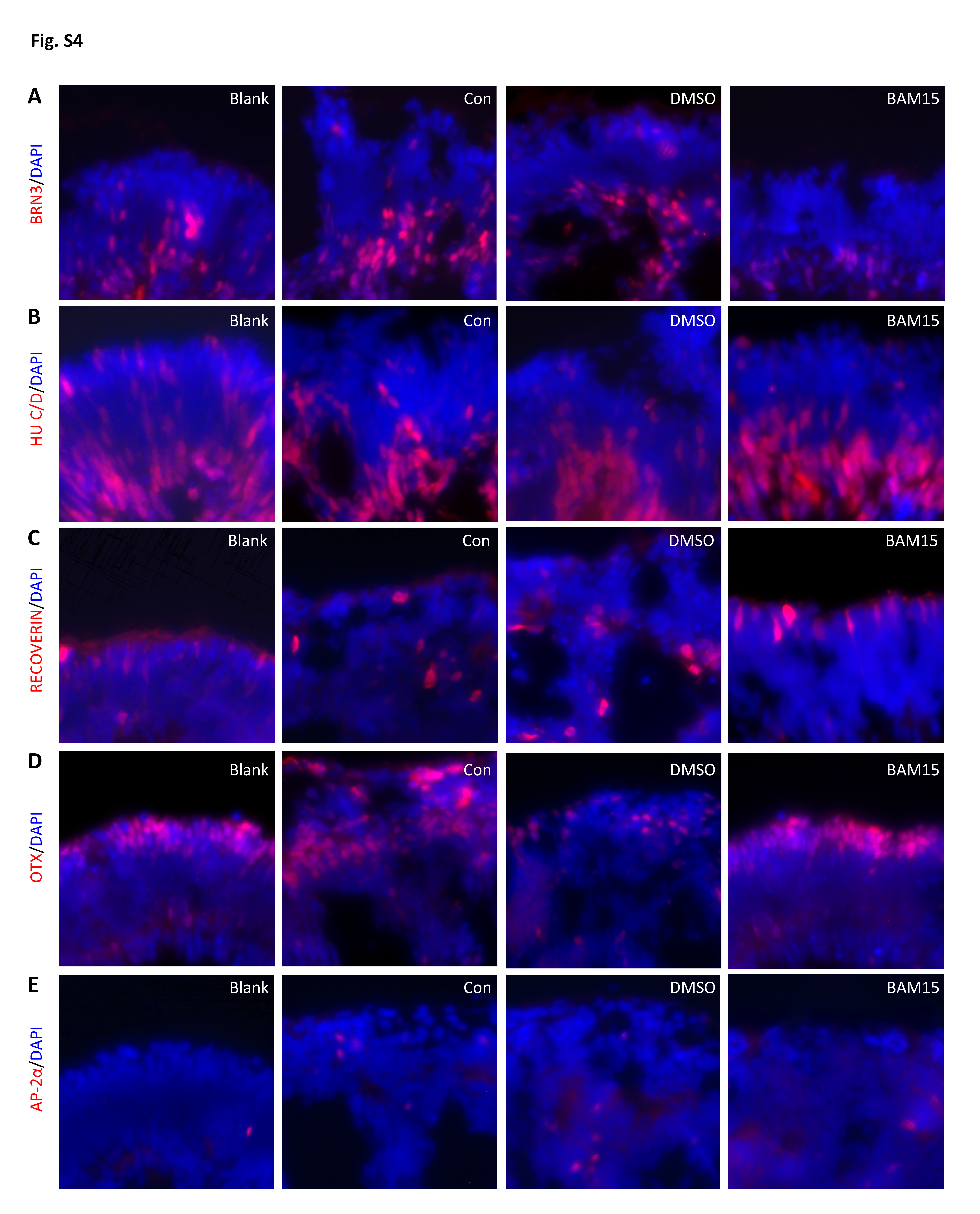

Supplement: Supplementary file 3 — Figure S4. Retinal cells were stained by immunofluorescence after 15 days of recovery in three-dimensional retinal tissues. Staining of ganglion cells (Brn3-/Hu C/D-positive, A-B), photoreceptor precursors (recoverin−/ OTX-positive, C-D), amacrine cells (AP2α-positive, E) was presented in different groups. (TIF 11747 kb) [file 13287_2019_1151_MOESM3_ESM.tif]

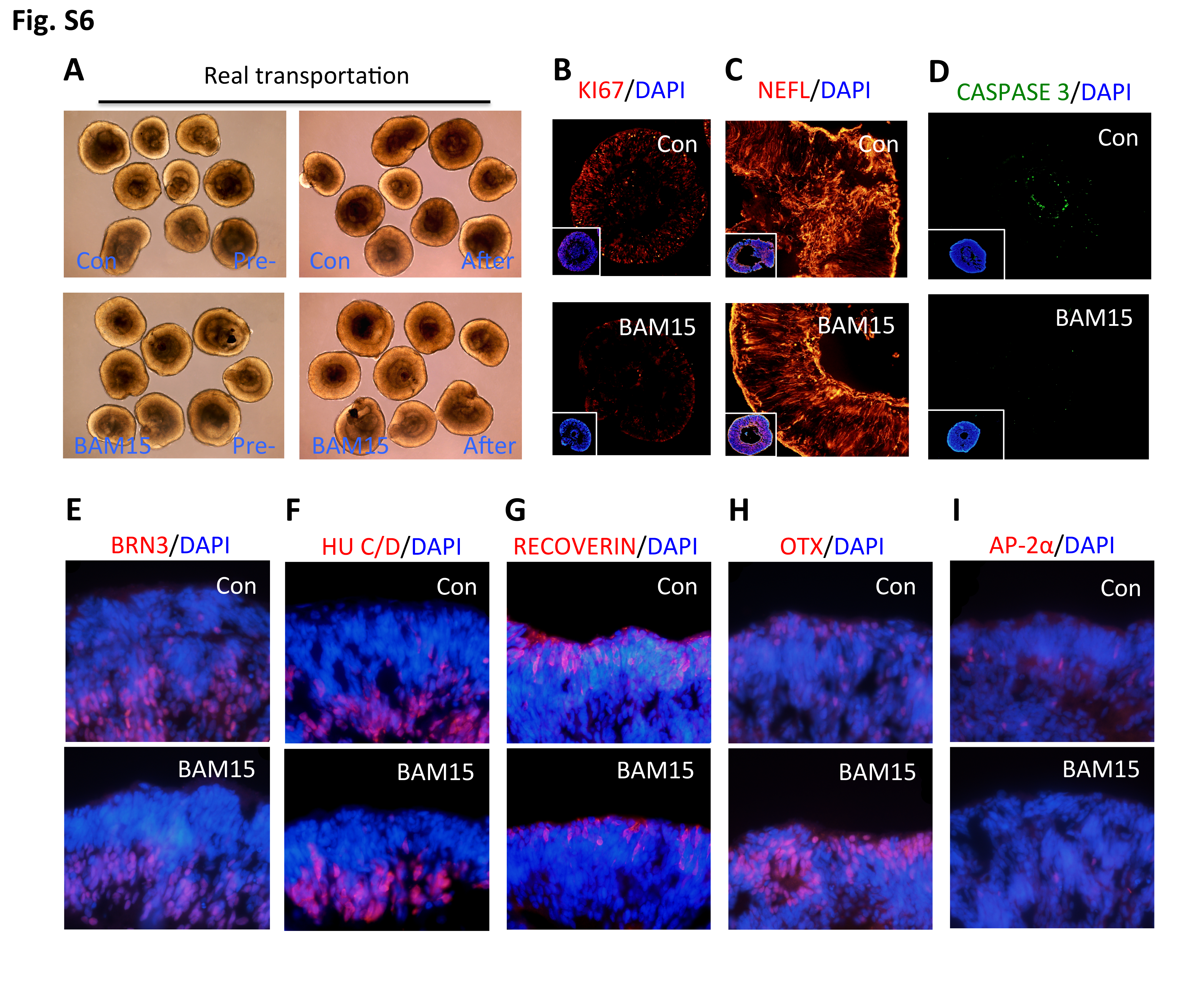

Supplement: Supplementary file 4 — Figure S6. In real transportation, trend of changes was similar to mimic transportation. (A) In the Con group and BAM15 group, morphology of retinal tissue varied slightly before and after transportation. (B-D) Immunofluorescence results presented the staining of Caspase-3 (B), NEFL(C) and Ki67(D) after real transportation. (E-I) Staining of ganglion cells (Brn3-/Hu C/D-positive, E-F), photoreceptor precursors (recoverin−/ OTX-positive, G-H), amacrine cells (AP2α-positive, I) was presented in the Con and BAM15 group. Scale bar = 50 μm (TIF 54539 kb) [file 13287_2019_1151_MOESM4_ESM.tif]

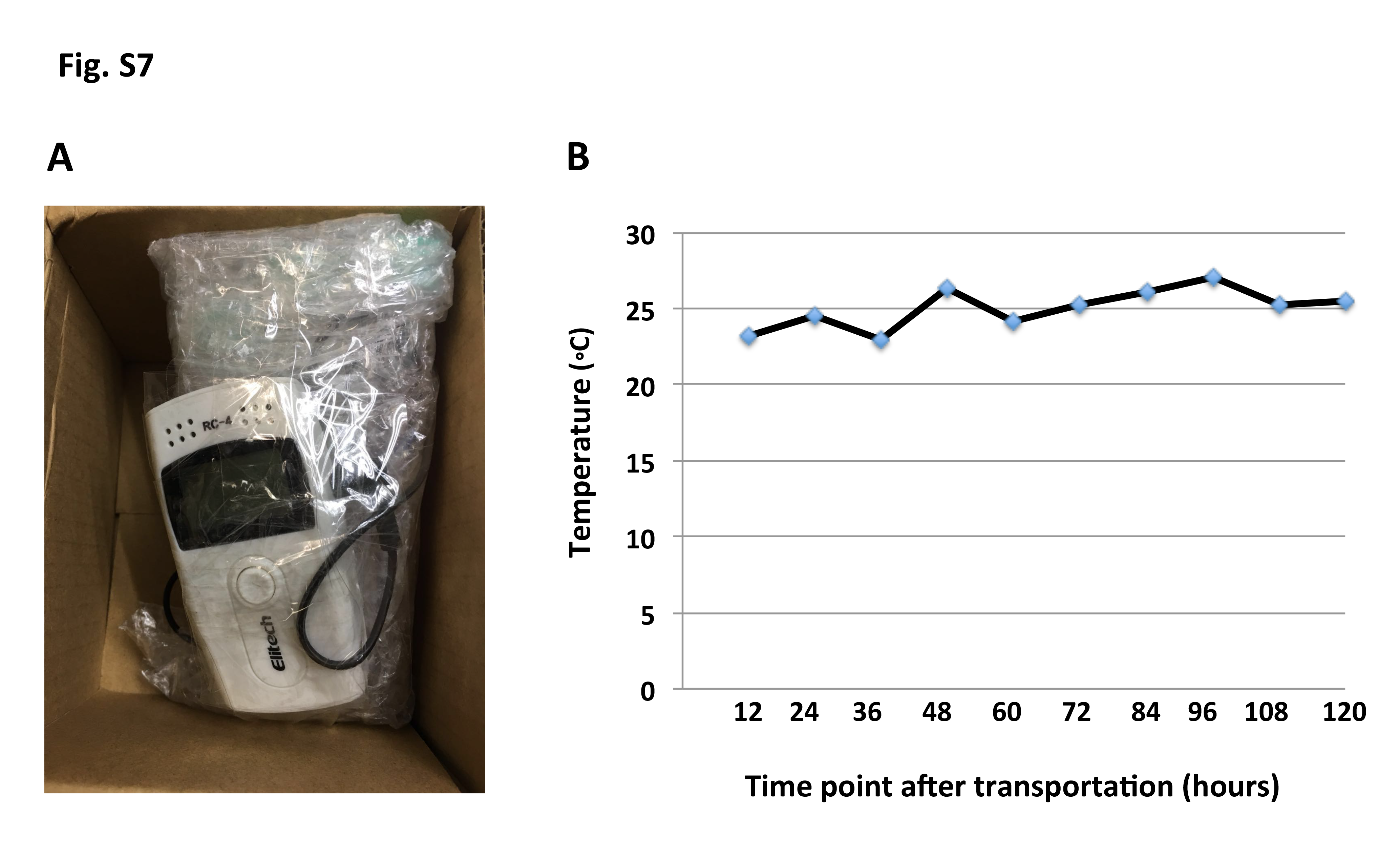

Supplement: Supplementary file 5 — Figure S7. The box used in real express transportation (A) and temperature at different time point during real transportation (B). (TIF 2677 kb) [file 13287_2019_1151_MOESM5_ESM.tif]

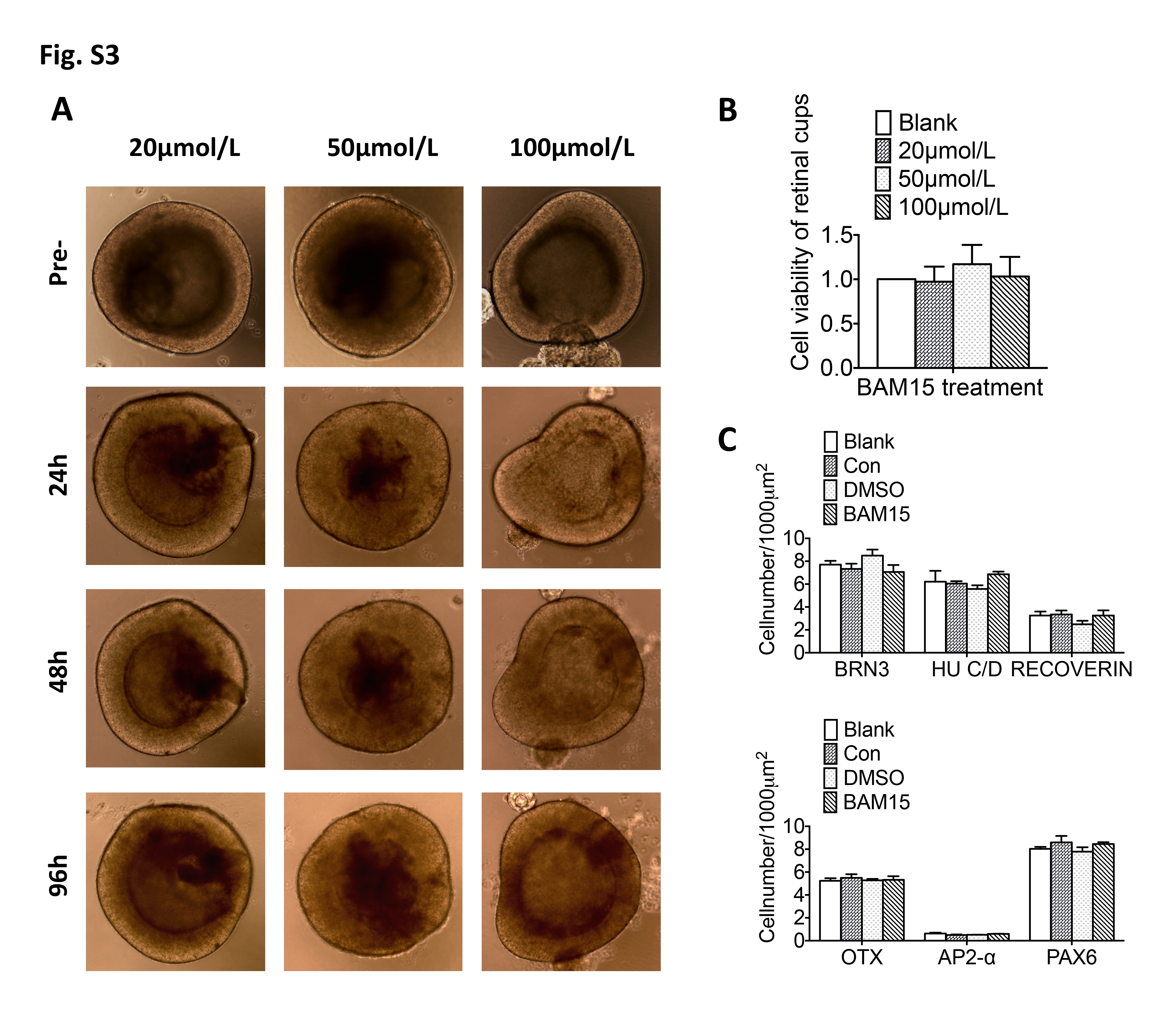

Supplement: Supplementary file 6 — Figure S3. Effects of BAM15 on cell proliferation and differentiation. (A) Outward appearance of retinal tissue with concentration at 20 μmol/L, 50 μmol/L and 100 μmol/L BAM15 in incubator at different time point. (B) BAM15 imposes little effects on cell proliferation in retinal organoid (blank, 1; con, 0.973 ± 0.139; DMSO, 1.122 ± 0.245; BAM15, 1.032 ± 0.180; p value > 0.5). (C) Quantification of retinal cell marker after transportation in Fig. 6 and Additional file 2: Figure S2D (Brn3+ cell/1000 μm2: blank, 7.70 ± 0.23, con, 7.32 ± 0.38, DMSO, 8.50 ± 0.42, BAM15, 7.06 ± 0.50; Hu C/D+ cell/1000 μm2: blank, 6.21 ± 0.78, con, 6.04 ± 0.17, DMSO, 5.58 ± 0.26, BAM15, 6.87 ± 0.19; recoverin+ cell/1000 μm2: blank, 3.26 ± 0.29, con, 3.35 ± 0.29, DMSO, 2.49 ± 0.25, BAM15, 3.25 ± 0.37; OTX+ cell/1000 μm2: blank, 5.24 ± 0.18, con, 5.50 ± 0.25, DMSO, 5.28 ± 0.10, BAM15, 5.32 ± 0.26; AP2α + cell/1000 μm2: blank, 0.63 ± 0.06, con, 0.49 ± 0.04, DMSO, 0.52 ± 0.01, BAM15, 0.59 ± 0.02; PAX+ cell/1000 μm2: blank, 8.03 ± 0.15, con, 8.59 ± 0.46, DMSO, 7.79 ± 0.31, BAM15, 8.46 ± 0.13). (TIF 7512 kb) [file 13287_2019_1151_MOESM6_ESM.tif]

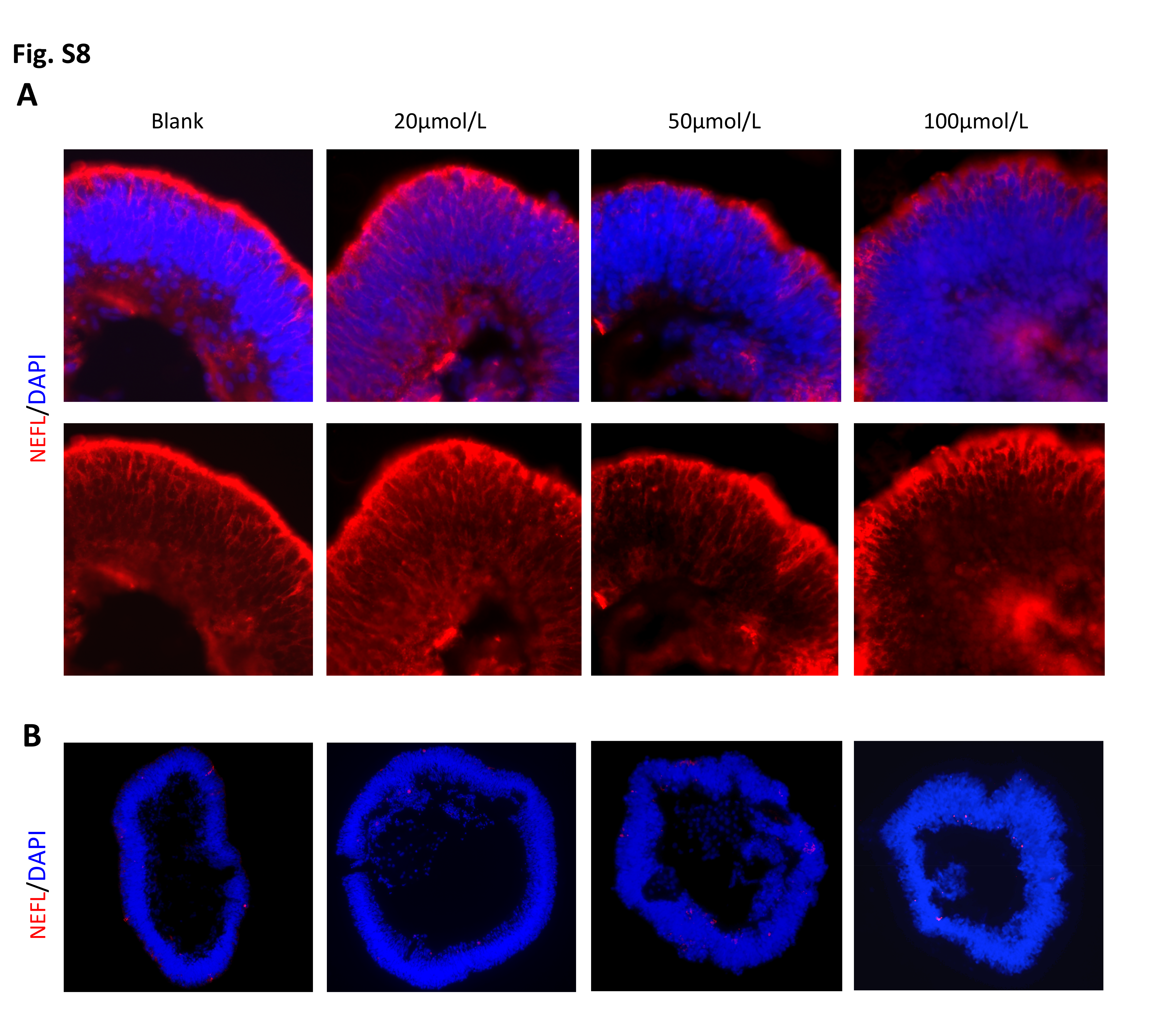

Supplement: Supplementary file 7 — Figure S8. Compared with the blank group, BAM15 at 20 μmol/L, 50 μmol/L and 100 μmol/L concentration imposes little effect on NEFL (A) and cleaved caspase-3 (B) expression in the incubator as negative control. (TIF 6721 kb) [file 13287_2019_1151_MOESM7_ESM.tif]

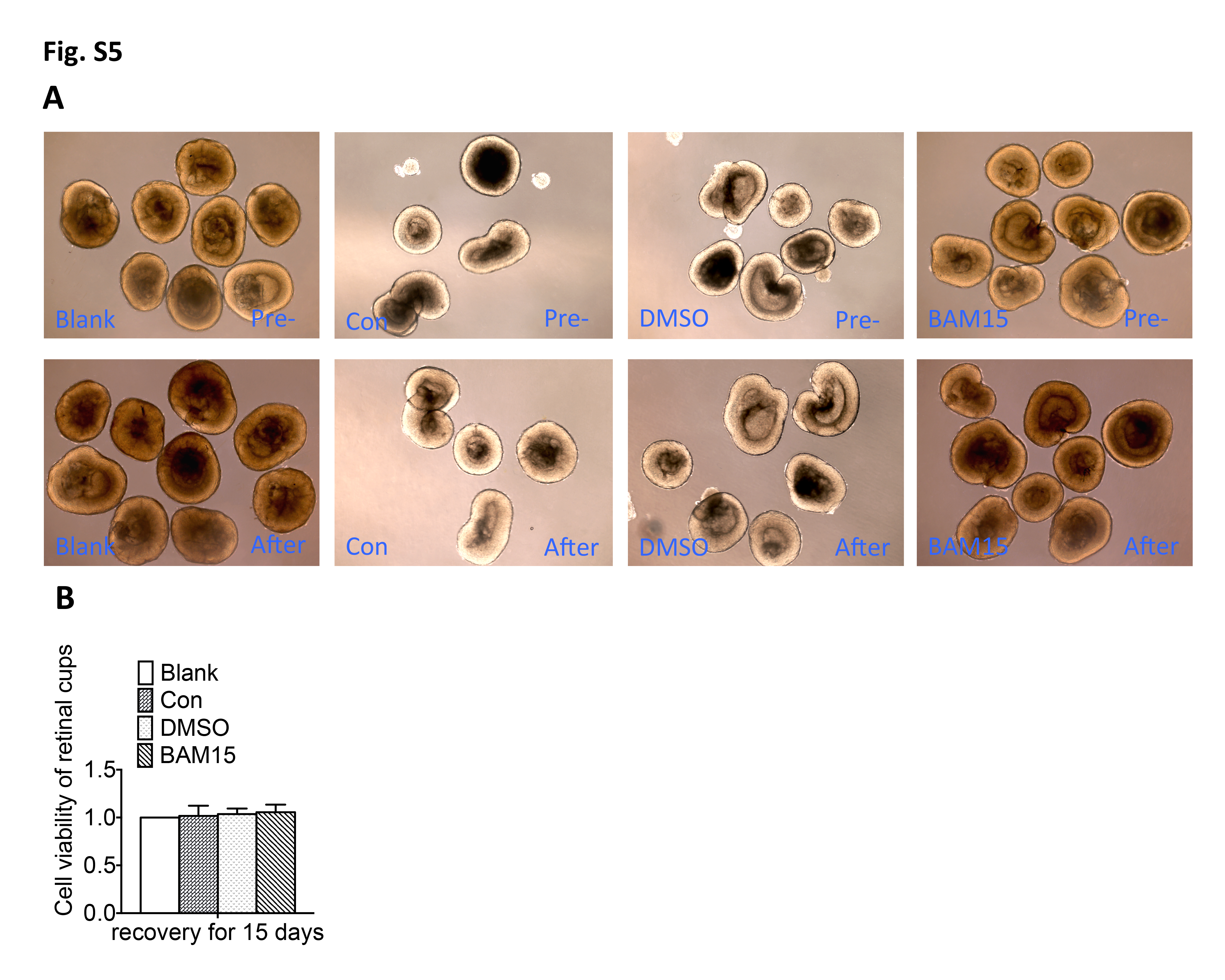

Supplement: Supplementary file 8 — Figure S5. (A) Outward appearance of retinal tissue after 5 days of transportation and 15 days of recovery. (B) Cell viability of retinal tissues after 5 days of transportation and 15 days of recovery (blank, 1; con, 1.02 ± 0.09; DMSO, 1.04 ± 0.05; BAM15, 1.05 ± 0.06; p = 0.40, 0.40, 0.37 respectively). (TIF 7675 kb) [file 13287_2019_1151_MOESM8_ESM.tif]
